# Green synthesis of silver nanoparticles mediated by *Solanum nigrum* leaf extract and their antifungal activity against pine pathogens

**DOI:** 10.1038/s41598-025-21291-0

**Published:** 2025-10-07

**Authors:** Waled Abd-Elhamed, Abeer A. Mohamed, Zakaria Hassan Saad, Shimaa El-Sayed Ibrahim Hassanien, Mohamed Z. M. Salem, Mervat EL-Hefny

**Affiliations:** 1 Agricultural Biochemistry Department, Faculty of Agriculture, Al-Azhar University, Cairo, 11823 Egypt; 2 Plant Pathology Research Institute, Agriculture Research Center (ARC), Alexandria, 21616 Egypt; 3 Department of Biochemistry, Faculty of Agriculture, Al-Azhar University, Nasr City, Cairo, 11751 Egypt; 4 Microbiology, Botany and Microbiology Department, Faculty of Science, Al-Azhar University (Girls’ Branch), Cairo, Egypt; 5 Forestry and Wood Technology Department, Faculty of Agriculture (El- Shatby), Alexandria University, Alexandria, 21545 Egypt; 6 Department of Floriculture, Ornamental Horticulture and Garden Design, Faculty of Agriculture (El-Shatby), Alexandria University, Alexandria, 21545 Egypt

**Keywords:** *Solanum nigrum* (L.), Green synthesis, Silver nanoparticles, Antifungal activity, Phytochemicals, Biochemistry, Biological techniques, Biotechnology, Microbiology, Plant sciences

## Abstract

Extracts from medicinal and aromatic plants have great benefits in controlling plant diseases. In this regard, the leaf aqueous extract (LAE) from the wild plant *Solanum nigrum* (L.) was used for the green synthesis of silver nanoparticles (AgNPs). The phytochemicals in the LAE were characterized by HPLC and FTIR analysis. The synthesized AgNPs were characterized using Transmission Electron Microscope (TEM), Energy Dispersive X-Ray (EDX), zeta potential, and FTIR Analysis. By HPLC analysis, the major compounds in the LAE were chlorogenic acid, gallic acid, syringic acid, and caffeic acid. The TEM analysis revealed that the average particle size ranged from 3.45 to 8.79 nm. The LAE and the synthesized AgNPs were evaluated for their antifungal activity against molecularly identified fungi *Fusarium circinatum*, *Phoma* sp., and *Pythium tardicrescens*, isolated from the diseased branches of the *Pinus halepensis* (Mill.) tree. At the LAE concentration of 1000 µg/mL, the fungal inhibition was reached 43.33%, 72.22%, and 37.40% against the growth of *P. tardicrescens*, *F. circinatum*, and *Phoma* spp., respectively. The synthesized AgNPs at 75 µg/mL showed fungal inhibition values of 58.14%, 56.66%, and 40.37% against *P. tardicrescens*, *F. circinatum*, and *Phoma* spp., respectively. Thus, the current study suggested producing stable, nontoxic, and eco-friendly Ag nanoparticles using the aqueous extract of *S. nigrum* leaves.

## Introduction

Plant-derived chemicals, from their botanical parts (leaves, seeds, stems, roots, fruits, etc.), even today, remain an important resource, especially in developing countries, to combat serious plant and human diseases^1–3^. These biochemicals are classified in several groups, like phenolic, flavonoids, essential oils, alkaloids, polysaccharides, fatty acids, and others^4–7^.

Black nightshade, or *Solanum nigrum* (L.), is an herbaceous plant that belongs to the nightshade family (Solanaceae). Originating in Eurasia, this species has spread to other continents^8,9^. The plant is notoriously hazardous, especially when it comes to the leaves and immature berries^10^. Immature green berries and leaves contain toxic compounds that can cause abdominal pain, vomiting, diarrhea, and potentially death in humans and animals. Ripe, black berries are generally considered safe to eat in limited quantities^11,12^. The plant contains several polyphenolic compounds, steroidal glycosides, steroidal saponins (diosgenin), steroidal genin (gitogenin), tannin, and glycoalkaloids (solanine, solamargine, solanigrine, and solasodine)^13–17^. Phenolic compounds are significant constituents of *S. nigrum*, and their presence may be responsible for some of its pharmacological actions^9^.

The bioactive compounds from *S. nigrum* can be used as bioreducing and capping agents for the green synthesis of several nanoparticles (NPs), making them nontoxic, cost-effective, and environmentally friendly. The green biosynthesis of ZnONPs using *S. nigrum* leaf extract was performed, suggesting potential applications in various medical and industrial fields^18,19^. The green synthesis of selenium NPs (SeNPs) by *S. nigrum* fruit extract demonstrated a notable dose-dependent decrease in free radicals, as well as antibacterial and anti-cancer properties^20^. The silver NPs (AgNPs) at different concentrations were assessed for their potential antibacterial effect against various nosocomial pathogens^21^. Inhibiting mycelial growth and conidial germination, breaking down cell walls and membranes, causing protein disruption, generating reactive oxygen species (ROS), affecting pathogen energy and substance metabolism, signal transduction, and genetic information processing are the main mechanisms by which AgNPs exhibit their antifungal properties^22–29^.

Pines are the most commercially significant trees in the world, particularly in the Mediterranean region, and the oldest stone fruit. Because young plants’ thin external protective tissues make them vulnerable to the entry of viruses, bacteria, nematodes, fungi, and viroids^30–34^. Diseases have a substantial impact on the output of approved planting material in nurseries. This means that both facultative and obligatory parasites infest planting material, which causes mass plant mortality^35,36^.

Thus, here, the aqueous extract from *S. nigrum* leaves and the biosynthesized AgNPs were used as antifungal agents against the isolated and molecularly identified fungi from diseased branches of *Pinus halepensis* (Mill.). The most important fungal diseases include the pinewood fungus, *Fusarium circinatum*, *Phoma* blight, and *Pythium tardicrescens*, the causal agents of pitch canker, *Phoma* blight, and Damping-off, respectively^37–39^. Plant diseases create intricate webs of interactions that make it challenging to develop management strategies^40,41^.

Therefore, the current study aimed to rapidly green manufacture AgNPs using the aqueous extract of *S. nigrum* leaves, explore the biomolecules that produce AgNPs, and then assess their antifungal properties.

## Materials and methods

### Preparation of the plant extract

**Fig. 1:**
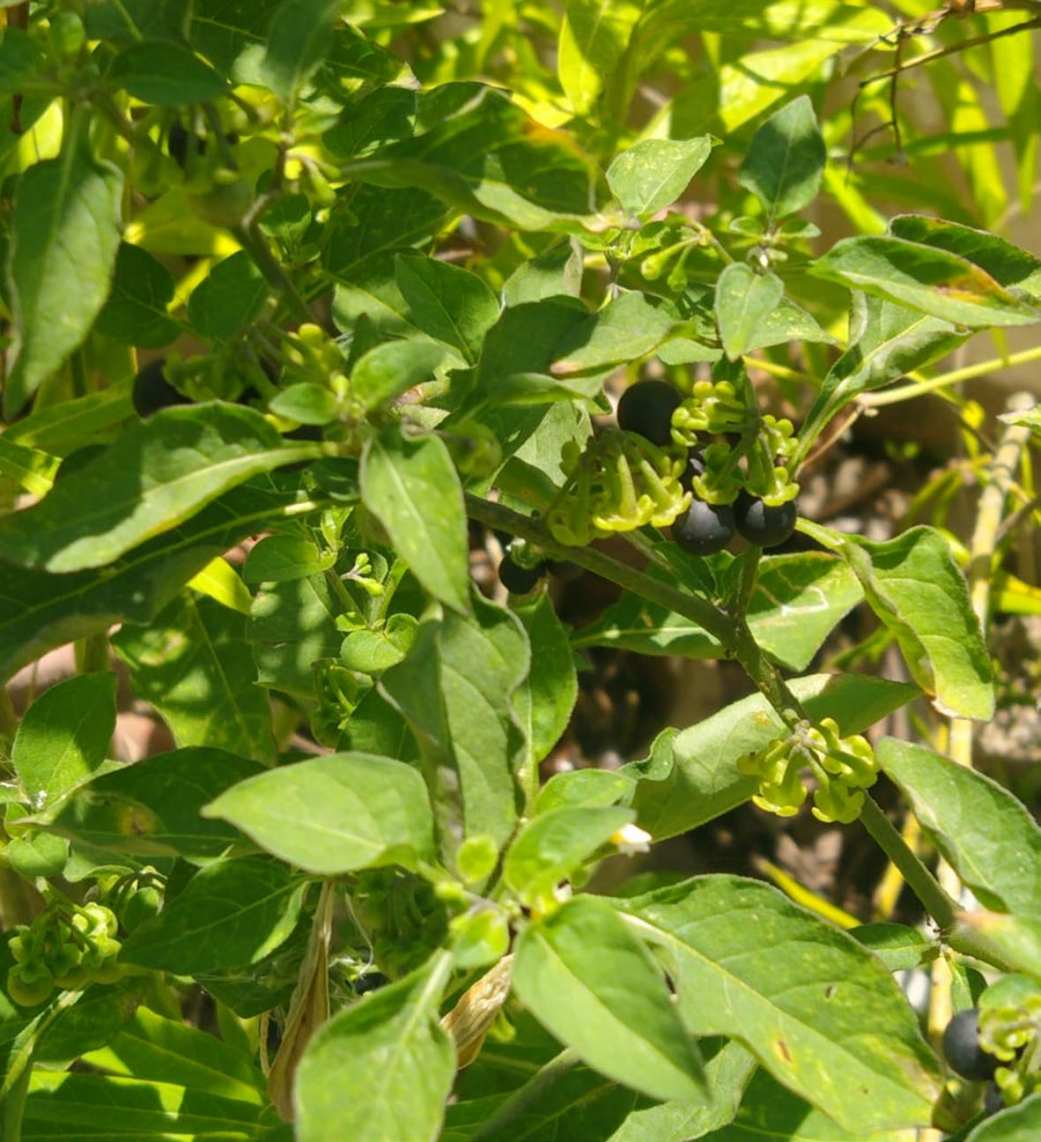
*Solanum nigrum* (L.) green leaves used for the water extraction.

This study has complied with relevant institutional, national, and international guidelines and legislation. This study does not contain any studies with human participants or animals performed by any of the authors in which the leaves of *Solanum nigrum* (L.) (Fig. 1) were collected from plants growing at the Nursery of the Department of Floriculture, Ornamental Horticulture, and Garden Design, Faculty of Agriculture, Alexandria University, Egypt. The plant with its voucher number Z0011 was identified by Dr. Mervat EL-Hefny (Department of Floriculture, Ornamental Horticulture and Garden Design, Faculty of Agriculture (El-Shatby), Alexandria University, Alexandria, Egypt. The plant was further identified and deposited at the Herbarium of the Plant Production Department, Faculty of Agriculture (Saba Basha), Alexandria University, Alexandria, Egypt^42^.

The gathered leaves were washed with tap water to remove any dust and dirt, and then air-dried at room temperature^43^. The dried leaves were ground into powder using a small laboratory mill. Approximately 10 g of *S. nigrum* leaf powder was combined with 100 mL of double-distilled water (ddW) and shaken for 2 h at 50 °C. The mixture was then filtered using Whatman filter paper No. 1 to obtain the *S. nigrum* leaf aqueous extract (LAE)^44^.

### HPLC conditions for phytochemical analysis

The HPLC analysis of *S. nigrum* LAE was carried out using an Agilent 1260 series device. The separation was performed using a Zorbax Eclipse Plus C8 column (4.6 mm × 250 mm, id, 5 μm film thickness). The mobile phase consisted of water (A) and 0.05% trifluoroacetic acid in acetonitrile (B) at a flow rate of 0.9 mL/min. A mobile phase linear gradient program was implemented with a step size of 1 min and durations of 5, 8, 12, 15, 16, and 20 min, using (A) concentrations of 82, 80, 60, 60, 82, 82, and 82%, respectively. The multi-wavelength detector was monitored at 280 nm. The injection volume was 5 µL for each sample solution (redissolved in acetone)^45^. The column temperature was maintained at 40 °C. Standard HPLC-grade phenolic and flavonoid compounds were used, including gallic acid, chlorogenic acid, catechin, methyl gallate, caffeic acid, syringic acid, pyrocatechol, rutin, ellagic acid, *p*-coumaric acid, vanillin, ferulic acid, naringenin, rosmarinic acid, daidzein, quercetin, cinnamic acid, kaempferol, and hesperetin. The identification of compounds was confirmed by comparing their retention time with the standard one. All chemical standards (high-performance liquid chromatography (HPLC grade) were from Sigma‒Aldrich (St. Louis, MO, USA)^44^.

### Preparation of silver nanoparticles

The biosynthesis of silver nanoparticles (AgNPs) using *S. nigrum* leaf aqueous extract (LAE) was adopted from the method of Bernardo et al.^46^ with minor modifications. 10 ml of *S. nigrum* LAE was combined with 90 ml of a 1 mM solution of AgNO_3_ (El-Gamhouria Trading Chemicals and Drugs Company, Alexandria, Egypt). The mixture was stirred for 30 min at a temperature between 60 and 70 °C. The observed change in color from green to brown is evidence that AgNPs have begun to appear in the samples. The solid mass of AgNPs was obtained through centrifugation (6000 rpm) for 10 min. The precipitated AgNPs were washed several times with ddW and dried overnight at 50 °C in a lab oven^47^.

### Characterization of the biosynthesized AgNPs

#### Transmission electron microscope (TEM) analysis

The morphological characteristics of the green-synthesized AgNPs were examined using a Transmission Electron Microscope (JEOL GEM-1400 plus) operated at 70 kV. For TEM imaging, a drop of the AgNP suspension was carefully placed onto carbon-coated copper grids and allowed to dry. Image analysis was performed using the ImageJ software^48^.

#### Energy dispersive X-Ray (EDX)

The confirmation of the presence of silver was confirmed via EDX. Using the Oxford 6587 INCA X-ray precision analyzer, an EDX microscopic examination was performed while the JEOL JSM-5500 LV electron microscope was scanned at a voltage of 20 kV^49^.

### The zeta potential

To assess the particle size distribution, the synthesized AgNPs were dispersed in deionized water and treated with ultrasonication to ensure proper homogenization. The suspension was then filtered and centrifuged at 5000 rpm for 10 min at 25 °C. The obtained supernatant was diluted 4 to 5 times and analyzed using a computer-controlled particle size analyzer (ZETA sizer nano series, Malvern Nano Zs)^50^.

### FT-IR analysis

The description of the functional groups of *S. nigrum* leaf aqueous extract (LAE) and the surfaces of AgNPs produced by *S. nigrum* LAE was carried out through FTIR analysis (Shimadzu device). In doing so, this was accomplished by carefully scanning using an FTIR type spectrum within a range of 4000–400 cm^− 1^ at a resolution of 4 cm^− 1^^51^.

### Antifungal activity

#### Fungal isolation

Diseased branch samples from *Pinus halepensis* (Mill.) were collected from the Alexandria Plant Protection Research Station. The disease samples were mainly collected from plants with typical *Phoma* blight. After cutting the samples into 0.5 × 0.5 cm pieces, they were surface sterilized in 75% ethanol for 1 min, washed with distilled water for 2 min, and then dried on sterile filter paper. A pure culture was then obtained by incubating the materials at 25 °C on potato dextrose agar (PDA; 200 g potato, 20 g dextrose, and 20 g agar/L) plates. Both single-tip and single-spore separation were used to produce pure cultures. At 4 °C, the purified isolates were maintained on PDA slants^52^.

#### Molecular identification

DNA extraction, PCR amplification, and sequencing of the isolate on PDA plates were performed for one week at 25 °C, and fresh mycelia were scraped off and collected in 1.5 mL centrifuge tubes. Then the genomic DNA was extracted^53,54^. Polymerase chain reaction (PCR) was carried out using the ITS region was amplified from the ITS1-5.8s and ITS2 regions using the universal primer pairs: ITS1/ ITS4^55^. The PCR results were evaluated using agarose gel electrophoresis after staining with ethidium bromide and sequenced at Macrogen, Scientific Services Company, Korea^3,56,57^. The sequences were edited with FinchTV v.1.4.0 (http://geospiza.com/finchtv). The sequence of DNA was subjected in GenBank on BLAST searches in the National Center for Biotechnology Information (NCBI) database using the Basic Local Alignment Search Tool (BLAST) version 2.15.0 (https://blast.ncbi.nlm.nih.gov/Blast.cgi) for preliminary identification of isolates.

#### Antifungal bioassay

Wood samples from *Fagus sylvatica* (L.) were prepared with a dimension of 2 × 2 × 0.7 cm. These wood samples were treated with or without the concentrated *S. nigrum* leaf aqueous extract (LAE) of 1000, 500, 250, and 125 µg/mL, which was prepared by dissolving in respective amounts of 10% dimethyl sulfoxide (10% DMSO) or the biosynthesized AgNPs at 75, 50, 25, 12, and 6 µg/mL, which was prepared by suspension in respective amounts of ddW. All the wood samples were subjected to antifungal evaluation against the growth of the three molds (*Phoma* sp., *F. circinatum*, and *P. tardicrescens*). For every concentration and isolated fungus, three replicates were taken from the wood samples. Each wood sample received 100 µL of the prepared concentrations of the LAE or AgNPs.

A 15-day-old PDA culture of each fungus was prepared. The treated wood samples with LAE or AgNPs, as well as the controls, were inoculated with each fungus disc (5 mm diameter) in a Petri dish containing 15 mL of PDA culture and then incubated for 14 days at 25 ± 1 °C^58,59^. The inhibition percentage of fungal growth (IPFG) = [(Control growth - Growth in treatment)/Growth in control] × 100, for the treated and untreated woods against each fungus, was measured and recorded using recommendations of previously published works^44,60^. The positive control, viz., Cure-M 72% WP (Mancozeb 64%+Metaxyl 8%), was tested at the recommended dosage (2 g/L) for antifungal activity by the poisoned food technique^61^. 10% DMSO was used as a negative control sample for LAE and ddW for AgNPs. The EC_50_ (The concentration causing 50% mycelia growth inhibition) for the extract and the AgNPs was calculated using the Probit analysis.

### Statistical analysis

The fungal inhibition percentages measured from the treated wood with the concentrated extract (1000, 500, 250, and 125 µg/mL) and the AgNPs (75, 50, 25, 12, and 6 µg/mL) were statistically analyzed using one-way ANOVA (analysis of variance) in SAS software (SAS Institute, Release 8.02, Cary, North Carolina, USA). The means from each treatment of the extract or the AgNPs were compared to the positive and negative control treatments using Duncan’s Multiple Range Test.

## Results and discussion

### Transmission electron microscope (TEM) analysis

**Fig. 2:**
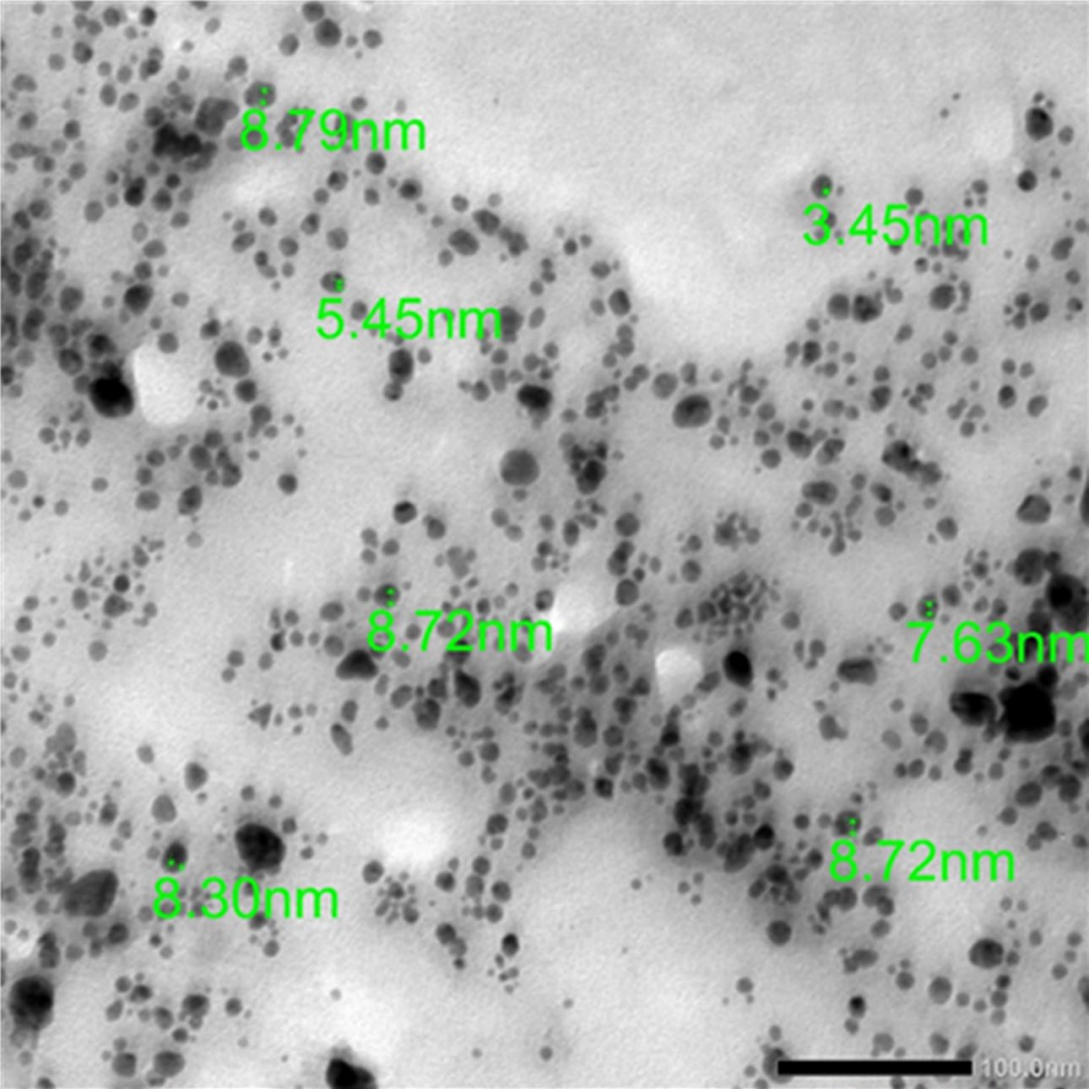
The TEM image for the particle size of the synthesized AgNPs.

The synthesis of silver nanoparticles (AgNPs) was confirmed via a green method using the leaf aqueous extract (LAE) of *Solanum nigrum* (black nightshade) through Transmission Electron Microscopy (TEM) analysis (Fig. 2). The TEM image reveals predominantly uniform, clearly spherical nanoparticles, well-distributed on the carbon-coated copper grid surface, with minimal agglomeration. This indicates the effectiveness of bioactive compounds present in *S. nigrum* LAE, such as phenolics, flavonoids, and alkaloids, in stabilizing and biologically capping the nanoparticles. The average particle size ranges between 3.45 and 8.79 nm, confirming their presence within the precise nanoscale range. This narrow size distribution reflects high efficiency in controlling the growth and formation process, which is attributed to the properties of the plant extract used. The small and homogeneous particle size provides a large surface area, making them suitable for various biomedical and applied fields such as antimicrobial agents, drug delivery, and catalysis. The scale bar in the image (100 nm) accurately depicts the particle size, further validating the analysis.

### Elemental analysis by EDX

**Fig. 3:**
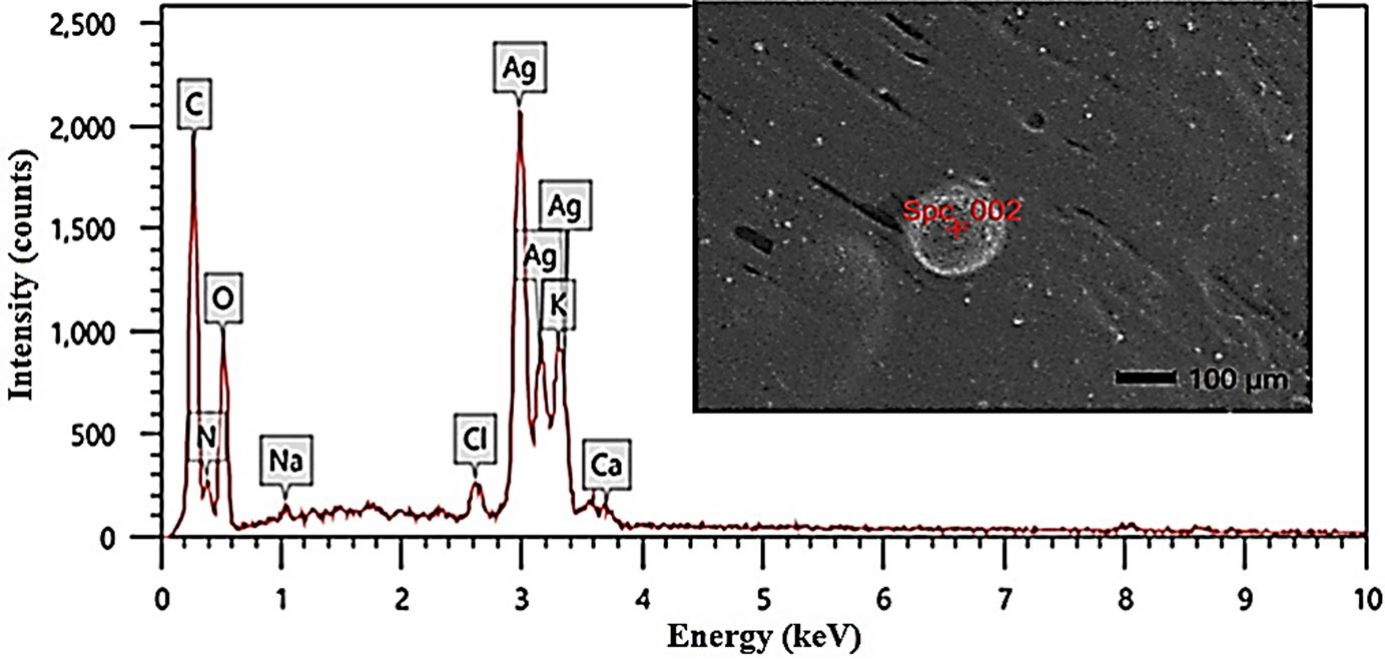
Elemental analysis of silver nanoparticles by EDX.

In Fig. 3, the SEM image coupled with EDX spectrum shows the surface morphology and elemental composition of the biosynthesized AgNPs using *S. nigrum* leaf aqueous extract (LAE). The mass and atomic percentages of the elemental composition from the green synthesized AgNPs from *S. nigrum* LAE are shown in Table 1. The mass percentages of C, N, O, and K were recorded as 25.06, 8.27, 29.75, and 4.95%, respectively. The mass% of Ag measured 30.20%. The Energy Dispersive X-ray (EDX) analysis confirmed the successful biosynthesis of AgNPs, as evidenced by the high silver content detected—30.20% by mass and 5.60% by atomic percentage.

**Table 1:** Elemental composition of silver nanoparticles biosynthesized using *Solanum nigrum* leaf aqueous extract, as determined by EDX analysis.

Element	Mass %	Atom%
C	25.06 ± 0.13	41.73 ± 0.21
N	8.27 ± 0.24	11.81 ± 0.35
O	29.75 ± 0.38	37.19 ± 0.47
Na	0.57 ± 0.05	0.50 ± 0.04
Cl	0.63 ± 0.03	0.36 ± 0.02
K	4.95 ± 0.09	2.53 ± 0.04
Ca	0.56 ± 0.04	0.28 ± 0.02
Ag	30.20 ± 0.25	5.60 ± 0.05
Total	100.00	100.00

### Zeta potential measurements

**Fig. 4:**
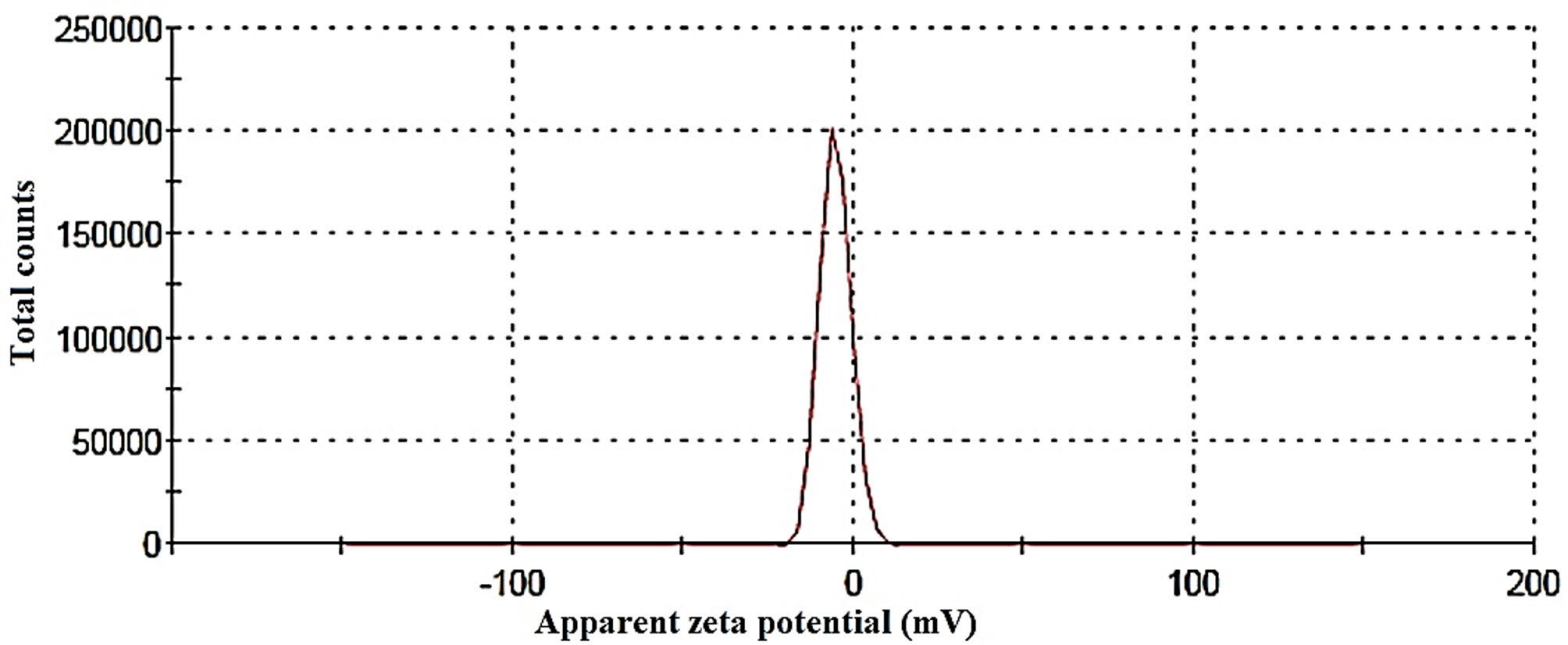
The apparent Zeta potential distribution of the synthesized AgNPs.

The zeta potential of the AgNPs synthesized using *S. nigrum* LAE (Fig. 4) was measured to be -5.4 mV, as shown in the zeta potential distribution graph. This value reflects the surface charge of the nanoparticles in the colloidal medium and serves as a key indicator of their colloidal stability. A zeta potential of -5.4 mV indicates that the AgNPs possess a slightly negative surface charge, resulting in weak electrostatic repulsion between particles. Consequently, the colloidal stability is considered low to moderate, as values below ± 10 mV are generally associated with a higher tendency for particle aggregation over time. In contrast, zeta potentials greater than ± 30 mV typically suggest strong repulsive forces and good colloidal stability.

#### FTIR spectral analysis of the extract and the produced AgNPs

**Fig. 5:**
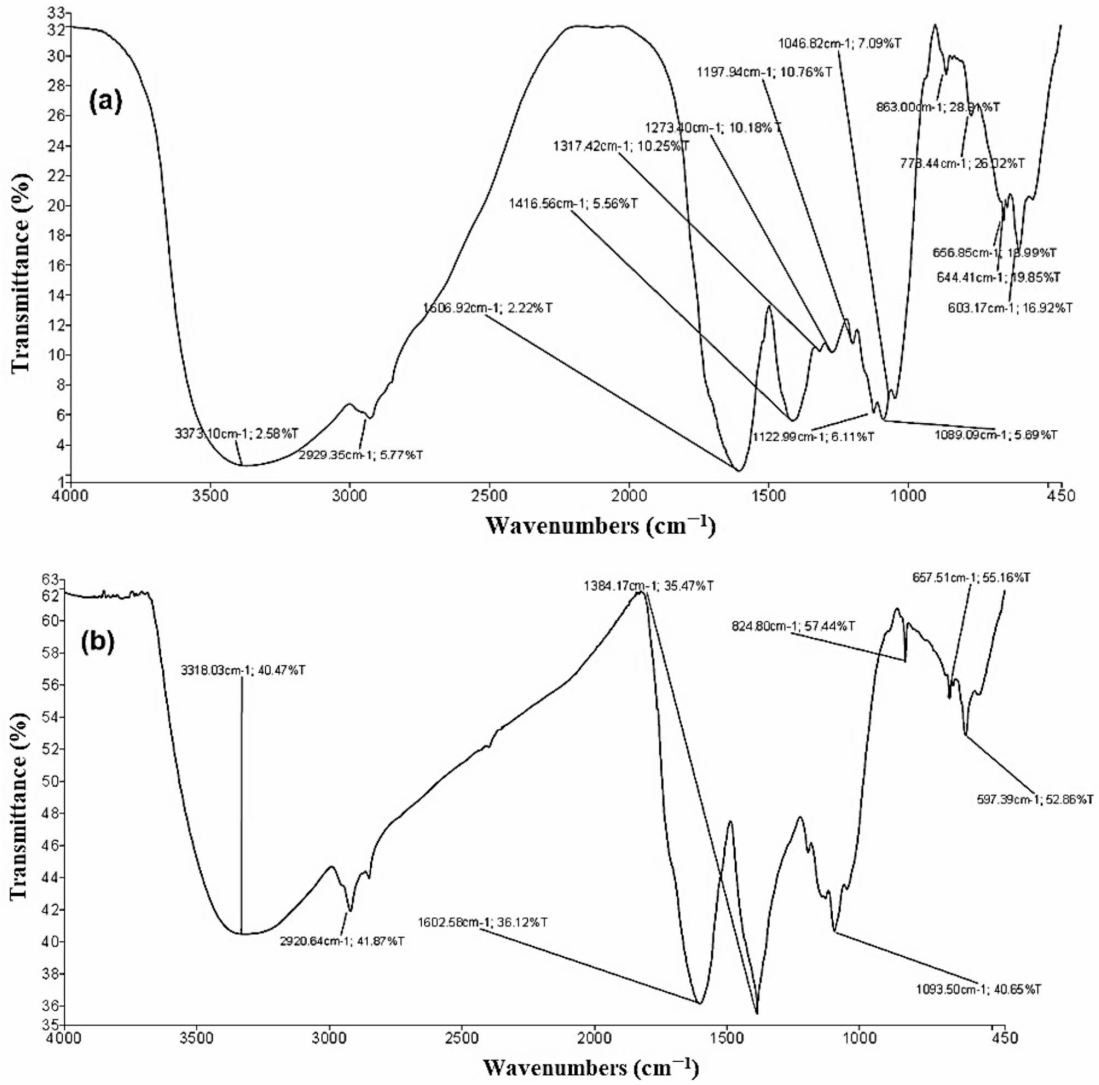
FTIR spectral analysis of the leaf aqueous extract from *S. nigrum* (a) and green-synthesized AgNPs (b).

The FT-IR spectra of the control leaf aqueous extract (LAE) from *S. nigrum* (before reaction without AgNO_3_ and the synthesized AgNPs (after reaction with AgNO_3_ are shown in Fig. 5a and b.

The FTIR spectral analysis of the extract (Fig. 5a) showed that the presence of a broad O–H/N–H stretch at 3373 cm^–1^ suggests an alcohol or amine. Additionally, the appearance of C–O or C–N stretches dominates the 1000–1300 cm^–1^ region. The band at about 1607 cm^–1^ and the out-of-plane C–H bends around 800–600 cm^–1^ indicate the aromatic or alkene-like structure.

Silver nitrate (AgNO_2_) is showing NO_2_^–^ stretching vibrations (e.g., ~ 1384 cm^–1^ for symmetric stretching). In Fig. 2b, peaks at 1384 cm^–1^ could be from nitrate or carboxylate groups, while peaks around 600–650 cm^–1^ (657.51 and 597.39 cm^–1^) might relate to metal-ligand vibrations, possibly involving Ag–X (X = N, O, S).

The FT-IR measurements were carried out to determine the biomolecules specifically bound to the silver particles involved in the reduction, capping, and stabilization. The present results showed sharp absorption peaks at wavenumbers of 3373.10, 2929.35, 1606.92, 1416.56, 1317.42, 1273.40, 1197.94, 1122.99, 1089.09, 1046.82, 863.00, 773.44 656.85, 644.41, and 603.17 cm^− 1^ for *S. nigrum* LAE prepared by the boiling method, and intense peaks were found at 3318.03, 2920.64, 1602.58, 1384.17, 1093.50, 824.80, 657.51, and 597.39 cm^− 1^ for AgNPs prepared by boiling method (Fig. 2a, b). The broad absorption band at 3373.10 cm^− 1^ in the IR spectra of *S. nigrum* LAE is indicative of the OH stretching of the H-bonded phenolic group^21^. The CH stretching of the methylene group may be the cause of the tiny, sharp band that appears at 2929.35 cm^− 1^^21,62^. The bands appearing at wavenumbers of 1089.09 cm^− 1^ and 1046.82 cm^− 1^ could be due to the C-O stretching of phenolic acid. The band at 824.80 cm^− 1^ was due to C–N stretching of aromatic phenols. The produced AgNPs exhibited the same absorption bands as those visible in the aqueous extract’s infrared spectrum.

### HPLC analysis of leaf extract

**Fig. 6:**
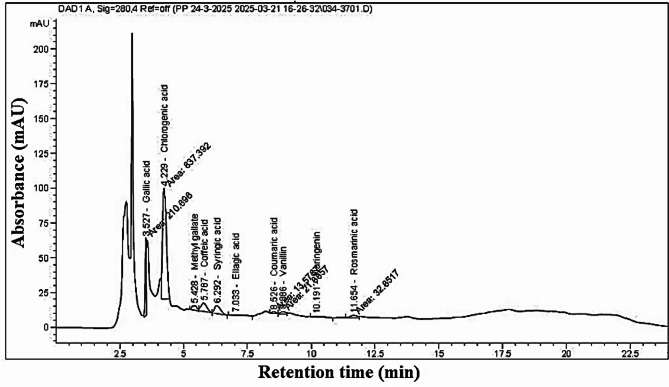
The HPLC chromatogram displays the peak area (mAU) of the chemical compounds identified in the aqueous extract from *Solanum nigrum* leaves.

Table 2; Fig. 6 show the chemical compounds identified in the leaf aqueous extract (LAE) from *S. nigrum*. The major compounds were chlorogenic acid (5834.41 µg/g), gallic acid (771.34 µg/g), syringic acid (269.32 µg/g), caffeic acid (250.91 µg/g), rosmarinic acid (159.53 µg/g), and methyl gallate (121.16 µg/g).

**Table 2:** HPLC analysis of leaf aqueous extract from *Solanum nigrum*.

RetTime [min]	Compound name	Area (%)	Area [mAU*s]	Conc. (µg/mL)	Conc. (µg/g)
3.527	Gallic acid	15.33	210.69	15.43	771.34
4.229	Chlorogenic acid	60.94	837.39	116.69	5834.41
5.428	Methyl gallate	3.15	43.31	2.42	121.16
5.787	Caffeic acid	7.12	97.81	5.02	250.91
6.292	Syringic acid	6.66	91.57	5.39	269.32
7.033	Ellagic acid	0.83	11.46	1.16	58.25
8.526	Coumaric acid	0.98	13.57	0.49	24.40
8.886	Vanillin	1.59	21.88	0.79	39.69
10.191	Naringenin	0.97	13.39	1.24	61.79
11.654	Rosmarinic acid	2.39	32.85	3.19	159.53

### Antifungal activity of aqueous extract and the synthesized AgNPs

**Fig. 7:**
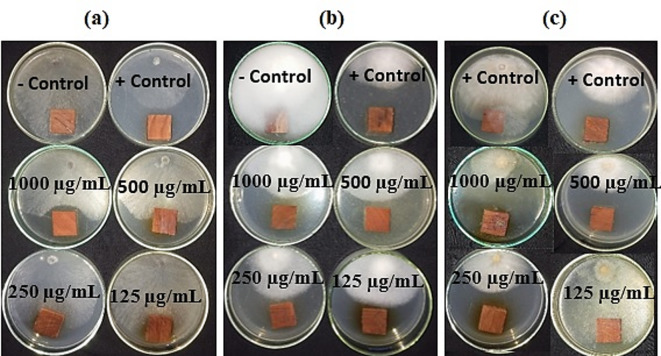
Antifungal activity of the aqueous extract from *Solanum nigrum* leaves. (a) *Phoma* spp., (b) *Pythium tardicrescens* (b), and *Fusarium circinatum* (c). +Control: Cure-M 72% WP (Mancozeb 64%+Metaxyl 8%); -Control: 10% DMSO.

The antifungal activity of *S. nigrum* leaf aqueous extract (LAE) against the growth of *Pythium tardicrescens*, *Fusarium circinatum*, and *Phoma* spp. is shown in Fig. 7; Table 3. At the LAE concentrations of 1000 and 500 µg/mL, the inhibition of fungal growth of *P. tardicrescens* was achieved at 43.33% and 40.00%, respectively, compared to the value recorded by the positive control (55.18%). The *S. nigrum* LAE at the concentrations of 1000, 500, 250, and 125 µg/mL showed fungal inhibition of *F. circinatum* with values of 72.22, 64.07, 52.96, and 50.37%, respectively, which are lower than the positive control (48.14%). At the LAE concentration of 1000 µg/mL, the highest fungal growth inhibition against *Phoma* spp. was reached (37.40%) compared to the positive control (36.29%).

**Fig. 8:**
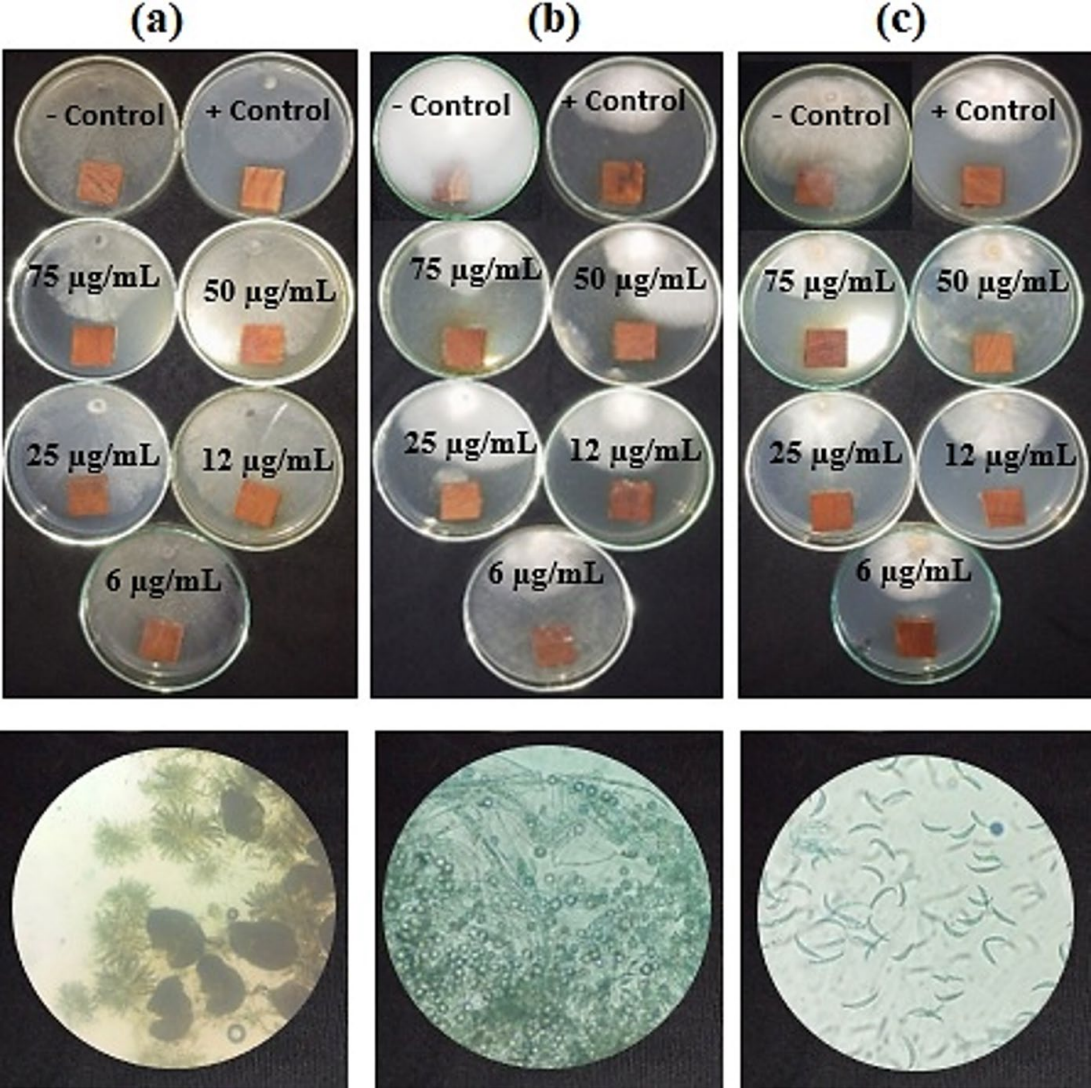
Antifungal activity of the green-synthesized silver nanoparticles by *Solanum nigrum* leaf aqueous extract. a) *Phoma* spp., (b) *Pythium tardicrescens* (b), and *Fusarium circinatum* (c). +Control: Cure-M 72% WP (Mancozeb 64%+Metaxyl 8%); -Control: ddW.

The antifungal activity of the treated wood samples with AgNPs synthesized using *S. nigrum* LAE is shown in Table 4; Fig. 8. At AgNPs concentrations of 75 and 50 µg/mL, there was fungal inhibition of 58.14% and 50.00%, respectively, against the growth of *P. tardicrescens*, compared to the positive control value (55.18%). The antifungal test against *F. circinatum* with AgNPs at 75, 50, and 25 µg/mL showed significant fungal inhibition of 56.66%, 54.44%, and 50.37%, respectively, compared to the positive control (48.14%). The highest antifungal activity against the growth of *Phoma* spp. by AgNPs was observed at 75 µg/mL, with a value of 40.37% compared to the positive control (36.29%). It should be mentioned that we were unable to detect any phytotoxicity or impact on the wood samples based on visual examination.

**Table 3:** Antifungal activity of the aqueous extract from *Solanum nigrum* leaves.

Extract concentration (µg/mL)	Fungal growth inhibition (%)		
	*Phoma* spp.	*Pythium tardicrescens*	*Fusarium circinatum*
1000	37.40a ± 8.63	43.33b ± 1.11	72.22a ± 1.11
500	21.48b ± 1.28	40.00c ± 1.11	64.07b ± 0.64
250	17.03b ± 1.69	37.03d ± 0.64	52.96c ± 0.64
125	2.96c ± 1.69	34.07e ± 1.69	50.37d ± 0.64
Negative control	0.00c	0.00f	0.00f
Positive control	36.29a ± 2.79	55.18a ± 0.64	48.14e ± 1.28

Values are means ± SD; Means with the same letter are not significantly different according to Duncan’s Multiple Range Test. Positive Control: Cure-M 72% WP (Mancozeb 64%+Metaxyl 8%); Negative Control: 10% DMSO.

**Table 4:** Antifungal activity of the synthesized silver nanoparticles from *Solanum nigrum* leaf aqueous extract.

AgNPs concentration (µg/mL)	Fungal growth inhibition (%)		
	*Phoma* spp.	*Pythium tardicrescens*	*Fusarium circinatum*
75	40.37a ± 1.28	58.14a ± 1.69	56.66a ± 1.11
50	12.22c ± 1.11	50.00c ± 1.11	54.44b ± 1.11
25	8.14d ± 2.79	44.44d ± 1.11	50.37c ± 1.69
12	3.33e ± 1.11	40.74e ± 1.69	47.41d ± 0.64
6	0.00f	2.96f ± 2.31	42.96e ± 0.64
Negative control	0.00f	0.00 g	0.00f
Positive control	36.29b ± 2.79	55.18b ± 0.64	48.14d ± 1.28

Values are means ± SD; Means with the same letter are not significantly different according to Duncan’s Multiple Range Test. +Control: Cure-M 72% WP (Mancozeb 64%+Metaxyl 8%); -Control: ddW.

The data presented in Table 5 compare the efficacy of *Solanum nigrum* leaf aqueous extract (LAE) and the green-synthesized AgNPs in inhibiting the growth of *Pythium tardicrescens*, *Fusarium circinatum*, and *Phoma* spp. The EC_50_ values from *S. nigrum* LAE indicate that the concentration required to achieve 50% inhibition of fungal growth of *P. tardicrescens*, *F. circinatum*, and *Phoma* spp. was found to be 2438.66, 232.96, and 1205.26 µg/mL, respectively. These values were lowered when the AgNPs were reduced to 48.82, 28.05, and 115.98 µg/mL. The higher EC_50_ indicates comparatively lower efficacy, while the lower values suggest comparable antifungal potency.

**Table 5:** The in vitro antifungal activity of *Solanum nigrum e*xtracts and green-synthesized AgNPs against *Pythium tardicrescens*, *Fusarium circinatum*, and *Phoma* spp.

Treatment	EC 50^a^ (µg/mL)	95% confidence limits		Slope^b^± SE	Intercept^c^±SE	(χ^2^)^d^	*R* ^2^
		Lower	Upper				
	*Pythium tardicrescens*						
*S. nigrum* LAE	2438.66	251.07	23686.56	0.41 ± 0.51	3.63 ± 0.51	0.88	0.97
AgNPs	48.82	32.757	72.743	2.47 ± 0.08	0.80 ± 0.08	0.00	0.805
	*Fusarium circinatum*						
*S. nigrum* LAE	232.96	90.02	602.89	0.96 ± 0.21	2.72 ± 0.21	0.88	0.98
AgNPs	28.05	4.296	183.160	0.429 ± 0.42	4.37 ± 0.42	0.98	0.97
	*Phoma* spp.						
*S. nigrum* LAE	1205.26	743.73	1953.21	2.60 ± 0.11	-3.02 ± 0.11	0.15	0.94
AgNPs	115.98	68.308	196.925	2.23 ± 0.12	0.358 ± 0.12	0.021	0.78

EC 50^a^: The concentration causing 50% mycelia growth inhibition. ^b^: Slope of the concentration-inhibition regression line ± standard error (SE). ^c^: Intercept of the regression line ± SE. (χ^2^)^d^: Chi-square value. R^2^: The coefficient of determination. LAE: Leaf aqueous extract. AgNPs: Silver nanoparticles.

## Discussion

In the present work, the biosynthesized silver nanoparticles (AgNPs) using *Solanum nigrum* (L.) leaf aqueous extract (LAE) were confirmed by the color change and spectroscopic analyses. The results in this study are consistent both morphologically and quantitatively with those reported previously^63^. The color of the silver nitrate solution turned brown upon the addition of plant leaf extract, signifying the creation of silver nanoparticles. The AgNPs’ activation of surface plasmon excitation caused the color shift^64,65^.

The size and shape of nanoparticles play an important role in many of the medicinal applications. The size of the synthesized particles was found to be 3.45 and 8.79 nm by boiled *S. nigrum* LAE, which showed enhanced fungal inhibition percentage against the tested fungi compared to the leaf extract. The particle size range and spherical shape of the AgNPs synthesized via the green method using *S. nigrum* LAE are relatively lower than the previous works of 28 nm^21^, 10–50 nm^62^, 50–100 nm (average size of 56.6 nm)^66^, and 4–25 nm^63^. The smaller size of nanoparticles could be related to the bioactivities. Iron oxide nanoparticles with a particle size of 10–30 nm showed significant antimycotic activity against *Trichothecium roseum*,* Cladosporium herbarum*,* Penicillium chrysogenum*,* Alternaria alternata*, and *Aspergillus niger*^67^. Smaller spherical (9 nm) and quasi-spherical (21 nm) AgNPs showed 100% suppression of the tested fungus and bacteria, demonstrating the selective size and shape-dependent capabilities of AgNPs in their antifungal activities^68^. Additionally, Chen et al. documented the impact of AuNPs on mouse studies for sizes ranging from 8 to 37 nm and 3 to 100 nm. AuNPs with diameters between 3 and 100 nm were shown to be less hazardous than those with sizes between 8 and 37 nm. The employment of organic reducing and capping chemicals during the NP synthesis, as well as synthetic techniques, is responsible for the toxicity of AuNPs. Because of their decreased surface area, smaller AuNPs are obviously more hazardous^69^. AgNPs with diameters of 5–20 nm had a significant inhibitory effect on the growth of the pathogenic fungus *Trichosporon asahii*^70^. AgNPs with sizes ranging from 9 to 30 nm showed different antimicrobial activities^71^. CuONPs with a small size (5.8 ± 3.5 nm) had a high potential for developing a topical antifungal treatment against *Candida albicans*^72^.

The reproducibility and dependability of employing plant-based bioactive chemicals for the efficient production and stabilization of nanoparticles are further supported by this agreement in nanoparticle size distribution and form^73–75^. This consistency validates the approach and confirms that the bio-capping and reducing agents present in *S. nigrum* LAE play a crucial role in controlling nanoparticle formation, as similarly demonstrated in the cited works.

The confirmation of metallic silver in the synthesized product was further confirmed by the EDAX analysis, where a strong signal from the silver atoms in the nanoparticles and other signals from O and other atoms were attributed to proteins/enzymes present in *S. nigrum* LAE. The disparity between the mass and atomic percentages of silver is attributed to its relatively high atomic weight, which results in a substantial contribution to the overall mass despite a lower atom count. Additionally, the spectrum revealed significant amounts of C (25.06% mass, 41.73% atomic), O (29.75% mass, 37.19% atomic), and N (8.27% mass, 11.81% atomic), indicating the presence of abundant organic compounds^63^.

These are most likely derived from the phytochemicals in *S. nigrum* LAE, which functioned as both reducing and stabilizing agents during nanoparticle synthesis. Other elements detected, such as K (4.95%), Na (0.57%), Ca (0.56%), and Cl (0.63%), are presumably residual components from the plant matrix or minor inorganic impurities and are not expected to significantly influence the nanostructure. Overall, the elemental composition supports the efficacy of the plant extract as a green, eco-friendly medium for the synthesis of stable and well-defined AgNPs^76,77^.

The carbonyl groups found in proteins and amino acid residues have a greater capacity to bind metal ions, according to the analysis of FTIR studies^78^. This suggests that proteins may form metal NPs (i.e., capping of AgNPs) to stop agglomeration and stabilize the medium. This implies that biological molecules may be able to serve the dual purposes of stabilizing and forming AgNPs in the aqueous media. The primary components used in the production of AgNPs are the carboxyl (-C = O), hydroxyl (-OH), and amine (-NH) groups of leaf extracts^79^. Spectra at 1606.92 cm^− 1^ or at 1602.58 cm^− 1^ for the LAE or LAE + AgNPs were assigned to C = C stretching (aromatic or alkene) or N–H bending (if amine)^21^. The assigned spectrum in LAE at 1416.56 cm^− 1^ is related to CH₂ or CH₃ bending (alkane) or aromatic C–C bending^62^. Spectra of C–O stretching (alcohols, ethers), or possible C–N stretching, were observed at wavenumbers 1197.94, 1122.99, 1089.09, and 1046.82 cm^− 1^ for LAE and 1093.50 cm^− 1^ for AgNPs^21,62^. Furthermore, the presence of a nitrate ion was the cause of the sharp band that appeared in the AgNPs’ IR spectra at wavenumbers of 1384.17 cm^− 1^. These findings implied that the phytoconstituents—polyphenols and flavonoids—found in *S. nigrum* LAE capped or shielded the produced AgNPs. The phenolic groups participating in ion exchange reactions were located at wavenumbers of 1606.92, 1317.42, and 1416.56 cm^− 1^ regions for *S. nigrum* LAE.

The current data clearly show that the synthesis of AgNPs involves the use of amides, polyphenols, carboxyl groups, amino groups, and amino acids, which are found in *S. nigrum* ALE extract^75,80–82^. Terpenoids and tannins are surface-active compounds found in leaf extracts that help stabilize nanoparticles and lower metal ions in metal ion reactions^83,84^.

The relatively low value of zeta potential observed in this study may be attributed to the nature of the bioactive compounds present in the plant extract, such as phenolics and flavonoids, which might not provide sufficient capping or electrostatic stabilization^75,85,86^. Additionally, factors such as ionic strength or pH of the suspension during measurement may have influenced the result. Despite the limited zeta potential value, the sharp and narrow peak centered at -5.4 mV indicates a relatively uniform distribution of surface charge among the particles, which is favorable in terms of particle homogeneity. Therefore, while the synthesized AgNPs may be suitable for short-term applications, further modification of synthesis conditions or the use of additional stabilizing agents may be necessary to enhance their long-term colloidal stability for biomedical or industrial uses^50,87^. While in another study, the particles are negatively charged with a value of -23.5 mV and are moderately stable^62^.

The results of this study showed the presence of gallic acid, chlorogenic acid, methyl gallate, caffeic acid, syringic acid, ellagic acid, coumaric acid, vanillin, and rosmarinic acid in *S. nigrum* leaves extract as analyzed by HPLC. Gallic acid equivalent phenolic compounds content, as well as quercetin equivalent flavonoids content, were highest in the leaf extract of the *S. nigrum*, and that could be the reason behind the highest antioxidant activity of the leaf extract^88^. Leaf water extract identified the following phenolic and flavonoid compounds: gallic acid, protocatechuic acid, chlorogenic acid, gentisic acid, caffeic acid, luteolin, and apigenin, with concentrations of 0.04, 0.19, 2.01, 1.81, 0.42, 0.8, and 0.12 mg/g of dry weight, respectively^9^. Numerous chemicals found in *S. nigrum* are what cause the bioreduction. Gallic acid, catechin, protocatechuic acid, caffeic acid, epicatechin, rutin, naringenin, and other polyphenolic chemicals are among its active constituents, along with glycoalkaloids, glycoproteins, and polysaccharides^89–91^. By HPLC analysis, the leaves were found to be richer in polyphenols of gentisic acid, luteolin, apigenin, kaempferol, and m-coumaric acid^92^. Numerous polyphenolic components, primarily flavonoids and steroids, have been found in the plant’s extract; further chemical elements found in the leaves include riboflavin, nicotinic acid, vitamin C, β-carotene, citric acid, and oils^93,94^.

Pines are prone to a wide range of diseases brought on by numerous pathogens such as *Fusarium circinatum*, *Phoma* sp., and *Pythium tardicrescens* (Accession numbers PV636492, PV892735, and PV636491), respectively. The *Phoma* sp. fungal isolate on pine trees was isolated in this work were identified using molecular analysis and a DNA sequence from the internal transcribed spacer (ITS) region. The sequence analysis revealed a 99% identity with *Phoma* sp., confirming the fungal pathogen. The efficacy of some plant extracts against the three fungi under study was evaluated.

For the antifungal activities against pathogens associated with the disease observed in *Pinus halepensis*, both extract and AgNPs were observed to have potential antifungal activities against the growth of *Phoma* spp., *Pythium tardicrescens*, and *Fusarium circinatum*. According to the EC_50_, the activity was achieved at lower concentrations with the synthesized AgNPs applied to wood samples and incubated with the fungal inoculum. Extracts from leaves of *S. nigrum* prepared using crude solvents exhibited higher antifungal activity against *A. niger*, *A. flavus*, and *C. albicans* as compared to their corresponding aqueous extracts. No good activity was observed in the aqueous extract. The pathogen-inhibiting activity was found to be dose-dependent^95^. Higher potential antibacterial action against all fungal forms was demonstrated by *S. nigrum*^96,97^. The fact that *S. nigrum* inhibited mycelial weight further demonstrated its effectiveness. Minimal inhibitory effects were observed in both the aqueous (1%) and crude extract (3%) for *Candida albicans*.

These possible bioactivities could be related to the presence of some bioactive compounds in the extract, like chlorogenic, gallic, and syringic acids. Against some fungal pathogens, the chlorogenic acid exhibited in vitro antifungal activity, resulting in decreased cell viability, an increased risk of mitochondrial depolarization and reactive oxygen species production, DNA fragmentation, and phosphatidylserine externalization, all of which are signs of an apoptotic process^98,99^. Gallic acid produced reactive oxygen species, externalized phosphatidylserine, and changed the membrane integrity and mitochondrial transmembrane potential of fungi^100^. Gallic acid can bind to the carbonic anhydrase protein active sites in both *Candida auris* and *Candida albicans*, influencing their catalytic activity, according to in silico research^101^. Several biological targets, including proteins, growth factors, transcriptional factors, and signaling molecules implicated in the development of disease, can have their dynamics altered by syringic acid^102^. Syringic acid inhibits the mycelial growth of *Botrytis cinerea*, with cell wall damage observed^103^.

The synthesized AgNPs by *S. nigrum* LAE showed potential antifungal activity at lower concentrations. At 500 µg/mL, the biosynthesized AgNPs showed encouraging antifungal activity against the four most prevalent *Aspergillus* species: *Aspergillus niger*, *A. terreus*, *A. flavus*, and *A. fumigatus*^104^. AgNPs at 50 µg/mL exhibited excellent antifungal efficacy against *A. niger*, *A. fumigatus*, and *F. soleni*^105^. At a concentration of 47–51 µg/mL, AgNPs had notable antifungal effectiveness against the mycotoxigenic fungi *A. flavus* and *A. ochraceus*^106^. At 100 µg/mL, biogenic AgNPs derived from *Syzygium cumini* leaf extract have antifungal efficacy against both *A. flavus* and *A. parasiticus* strains^107^. In an in vivo trial, 20 ppm of AgNPs lowers the relative vascular discoloration and the severity of *Verticillium* wilt by 97% and 87%, respectively, in comparison to the infected and untreated control plants of eggplants (*Solanum melongena* L.)^108^.

AgNPs treatment against several pathogens showed different mechanisms of action. AgNPs have been utilized extensively for plant disease control in recent years due to their high efficiency, broad-spectrum antibacterial action, low resistance, and exceptional safety^104,109,110^. AgNPs have also been linked to a number of antimicrobial mechanisms, particularly in bacteria. These mechanisms include damaging DNA, cell walls, and cell membranes; blocking electron transport or ATP biosynthesis; interfering with protein synthesis; and triggering the production of reactive oxygen species^111^. AgNPs’ toxicological impact and antifungal molecular mechanisms are yet unknown, particularly concerning their effects on important biological processes and intracellular signaling pathways.

In the pathogens *Alternaria brassicicola* and *Fusarium oxysporum*, biosynthesized AgNPs produced by the cell-free filtrate of *Trichoderma viride* (MTCC 5661) cause osmotic imbalance, produce superoxide radicals, and damage cellular integrity^112^. At an EC_50_ value of 2.0 µg/mL, two nm-AgNPs nearly eliminated the 100% *pathogenicity of Magnaporthe oryzae*. Subsequent research has shown that treatment with AgNP reduces the formation of appressoria and alters the shape of cell walls. The decreased phosphorylation level of the MAPK MoPmk1 and compromised conidial autophagy were the causes of a decrease in appressorium formation following AgNP therapy^113^. AgNPs with a diameter of 20–30 nm or biosynthesized AgNPs can stop the rice blast fungus *M. oryzae* from growing its hyphal and appressorium^114^. AgNPs at 75 ppm successfully suppressed the spore germination and mycelial growth of four kiwifruit rot pathogens: *Botryosphaeria dothidea*, *Alternaria alternata*, *Diaporthe actinidiae*, and *Pestalotiopsis microspora*. Furthermore, AgNPs made the cell membrane of the mycelium more permeable, which suggested that intracellular material was leaking out^115^.

Highly reactive AgNPs smaller than 5 nm were found to have strong antifungal action against *Candida parapsilosis*, *Candida glabrata*, *Candida guillermondii*, and *Candida albicans*. These findings hold great promise for the development of alternative treatments for fungal diseases in plants, animals, and humans, as well as for coating traditional surgical surfaces^116^.

While there are numerous inherent benefits to using nanoparticles as bioactive agents, issues including toxicity at high concentrations and hazardous environmental disposal may prevent their widespread use, which presents new research opportunities. An additional drawback is that using nanoparticles is challenging due to their environmental stability. The stability of the suspension is significantly influenced by the particle size and its affinity for other environmental elements. Metal nanoparticles are more prone to oxidation in the air due to their lower stability in nature. Since these nanoparticles cannot be stored in normal ambient conditions for future use, they are maintained in a specific environment of inert gases^117–119^.

The limitation of this study can be drawn in the need for more research and studies in the future. It is crucial to remember that data on bioactivity measured in a lab setting do not always correspond to toxicity in vivo. This study, therefore, paves the way for additional investigation into the effects of plant extracts and nanoparticles over a longer time frame, or shelf life, which may be utilized as alternatives to synthetic fungicides. Our findings regarding the antifungal properties may result in improved formulations for wood protection and agricultural uses.

## Conclusion

The study concludes that *S. nigrum* leaf extract-stabilized silver nanoparticles could be easily and rapidly synthesized by a green approach. The structure, morphology, and size (dimension) of prepared AgNPs were examined by FT-IR, zeta potential, SEM-EDX, and TEM analysis. The average diameter of the NPs lies between 3.45 and 8.79 nm, as confirmed by TEM study. The FT-IR spectra revealed the functional groups of stretching bands for AgNPs. The synthesized NPs were studied for the antifungal activities when applied to wood samples against the molecularly identified fungi *Pythium tardicrescens*, *Fusarium circinatum*, and *Phoma* spp. Finally, the present study is so helpful and useful to the scientific community for using the AgNPs as potent applications to the compact fungal diseases of *Pinus halepensis* or when applied to wood samples.

## Data Availability

All data generated or analyzed during this study are included in this published article.
